# Supplementary material for: A comparison of risk factors for cryptosporidiosis and non-cryptosporidiosis diarrhoea: A case-case-control study in Ethiopian children
Source: PLoS Negl Trop Dis. 2022 Jun 6;16(6):e0010508. doi: 10.1371/journal.pntd.0010508 (PMC9203008; doi:10.1371/journal.pntd.0010508)
Supplement: S1 Checklist — (PDF) [file pntd.0010508.s001.pdf]

STROBE Statement—Checklist of items that should be included in reports of ***case-control studies***

	Item No	Recommendation	Where addressed
**Title and abstract**	1	(*a*) Indicate the study's design with a commonly used term in the title or the abstract	Abstract paragraph 2
		(*b*) Provide in the abstract an informative and balanced summary of what was done and what was found	Abstract paragraph 2 and 3
**Introduction**			
Background/rationale	2	Explain the scientific background and rationale for the investigation being reported	Paragraph 2
Objectives	3	State specific objectives, including any prespecified hypotheses	Paragraph 3
**Methods**			
Study design	4	Present key elements of study design early in the paper	Introduction paragraph 3; Methods "Study design"
Setting	5	Describe the setting, locations, and relevant dates, including periods of recruitment, exposure, follow-up, and data collection	"Study design" and "Selection of cases and controls" – paragraph 1-3
Participants	6	(*a*) Give the eligibility criteria, and the sources and methods of case ascertainment and control selection. Give the rationale for the choice of cases and controls	"Study design" and "Selection of cases and controls" – paragraph 1-3
		(*b*) For matched studies, give matching criteria and the number of controls per case	• "Selection of cases and controls" – 1st paragraph<br>• S1 Appendix Supplementary methods : "Recruitment of non-diarrhoea controls"
Variables	7	Clearly define all outcomes, exposures, predictors, potential confounders, and effect modifiers. Give diagnostic criteria, if applicable	• Outcome and exposure variables: "Data collection" and S1 Appendix p3 "Variables".<br>• Confounders: "Statistical methods" 2nd paragraph and S1 Appendix Supplementary Methods: "Modelling strategy and statistical methods"

Data sources/ measurement	8*	For each variable of interest, give sources of data and details of methods of assessment (measurement). Describe comparability of assessment methods if there is more than one group	• "Data collection"<br>• S1 Appendix p3 "Variables"<br>• S1 Appendix Table A
Bias	9	Describe any efforts to address potential sources of bias	• Methods – statistical methods – paragraph 2<br>• Selection bias due to missing outcome: Section p8 in S1 Appendix<br>• Differential recall bias: S1 Appendix – Variables – paragraph 2<br>• Selection bias: Discussion paragraph 8,<br>• Overadjustment bias: Discussion paragraph 4<br>• Discussion paragraphs 5-11 all address potential sources of bias<br>• Section in S1 Appendix: Bias analysis: Missing outcome for diarrhoea cases<br>• Section in S1 Appendix: Bias analysis: Enrolment of controls from vaccination rooms and by household visit<br>• Section in S1 Appendix: Bias analysis: Differential exposure misclassification for the sanitation variable
Study size	10	Explain how the study size was arrived at	"Selection of cases and controls" and S1 Appendix: "Sample size considerations" p5
Quantitative variables	11	Explain how quantitative variables were handled in the analyses. If applicable, describe which groupings were chosen and why	• "Data collection"<br>• S1 Appendix p3 "Variables"<br>• S1 Appendix Table A
Statistical methods	12	(*a*) Describe all statistical methods, including those used to control for confounding	• Methods - "Statistical methods"<br>• S1 Appendix: "Modelling strategy and statistical methods"
		(*b*) Describe any methods used to examine subgroups and interactions	• S1 Appendix "Modelling strategy and statistical methods" 7th paragraph<br>• S1 Appendix: "Bias analysis: Enrolment of controls from vaccination rooms and by household visit" 2nd paragraph

			• "Statistical methods"; last two sentences
		(*c*) Explain how missing data were addressed	• Missing exposure values: "Statistical methods" 1st paragraph<br>• Missing outcome variable: Section in S1 Appendix: "Bias analysis: Missing outcome for diarrhoea cases"<br>• Table A in S1 Appendix
		(*d*) If applicable, explain how matching of cases and controls was addressed	• "Selection of cases and controls"<br>• Frequency-matching procedure further detailed in S1 Appendix "Supplementary methods Recruitment of non-diarrhoea controls"
		(*e*) Describe any sensitivity analyses	• "Table D: Selection bias adjusted crude odds ratios, under 2-year-olds" in S1 Appendix "Bias analysis: Missing outcome for diarrhoea cases"
**Results**			
Participants	13*	(a) Report numbers of individuals at each stage of study—eg numbers potentially eligible, examined for eligibility, confirmed eligible, included in the study, completing follow-up, and analysed	Results 1st paragraph; Fig 2 flowchart
		(b) Give reasons for non-participation at each stage	Fig 2 flowchart
		(c) Consider use of a flow diagram	Fig 2 flowchart
Descriptive data	14*	(a) Give characteristics of study participants (eg demographic, clinical, social) and information on exposures and potential confounders	Results 2nd paragraph; Table 1
		(b) Indicate number of participants with missing data for each variable of interest	S1 Appendix: "Table A: Distribution of case and control subjects according to all exposures, with counts and proportions of missing values"
Outcome data	15*	Report numbers in each exposure category, or summary measures of exposure	• Table 2<br>• S1 Appendix: "Table A: Distribution of case and control subjects according to all exposures, with counts and proportions of missing values"
Main results	16	(*a*) Give unadjusted estimates and, if applicable, confounder-adjusted estimates and their precision	• Table 2, Table 3, Table 4

	(eg, 95% confidence interval). Make clear which confounders were adjusted for and why they were included	• S1 Appendix: "Table A: Distribution of case and control subjects according to all exposures, with counts and proportions of missing values"<br>• Confounder adjustment rationale: "Statistical methods" 2nd paragraph and S1 Appendix Supplementary Methods: "Modelling strategy and statistical methods"
	(*b*) Report category boundaries when continuous variables were categorized	• Table 2, Table 3, Table 4 includes category boundaries<br>• Details in S1 Appendix: "Supplementary methods – Variables" – 1st paragraph
	(*c*) If relevant, consider translating estimates of relative risk into absolute risk for a meaningful time period	Not relevant

Other analyses	17	Report other analyses done—eg analyses of subgroups and interactions, and sensitivity analyses	Various Bias analyses in S1 Appendix (see table of contents)
**Discussion**			
Key results	18	Summarise key results with reference to study objectives	Discussion paragraph 1 (acute malnutrition), 2 (previous healthcare visits), 3 (water source), 4 (socioeconomic and caregiver factors)
Limitations	19	Discuss limitations of the study, taking into account sources of potential bias or imprecision. Discuss both direction and magnitude of any potential bias	Discussion paragraphs 5-11
Interpretation	20	Give a cautious overall interpretation of results considering objectives, limitations, multiplicity of analyses, results from similar studies, and other relevant evidence	Discussion paragraph 11 (last)
Generalisability	21	Discuss the generalisability (external validity) of the study results	Discussion 1st paragraph
**Other information**			
Funding	22	Give the source of funding and the role of the funders for the present study and, if applicable, for the original study on which the present article is based	This work was supported by the Research Council of Norway [grant numbers 255571 and 223269 to H.S.]; Bill and Melinda Gates Foundation [grant number OPP1153139]; Norwegian Association for Medical Microbiology; University of Bergen and Vestfold Hospital Trust.

*Give information separately for cases and controls.

Note: An Explanation and Elaboration article discusses each checklist item and gives methodological background and published examples of transparent reporting. The STROBE checklist is best used in conjunction with this article (freely available on the Web sites of PLoS Medicine at http://www.plosmedicine.org/, Annals of Internal Medicine at http://www.annals.org/, and Epidemiology at http://www.epidem.com/). Information on the STROBE Initiative is available at http://www.strobe-statement.org.
