# Supplementary material for: A comparison of risk factors for cryptosporidiosis and non-cryptosporidiosis diarrhoea: A case-case-control study in Ethiopian children
Source: PLoS Negl Trop Dis. 2022 Jun 6;16(6):e0010508. doi: 10.1371/journal.pntd.0010508 (PMC9203008; doi:10.1371/journal.pntd.0010508)
Supplement: S1 Appendix — Table A in S1 Appendix. Distribution of case and control subjects according to all exposures, with counts and proportions of missing values. Table B in S1 Appendix. Hierarchical mediation analysis of risk factors for cryptosporidiosis and non-cryptosporidiosis diarrhoea, in children under 2 years old. Table C in S1 Appendix. Duration of diarrhoea, on enrolment, in cryptosporidiosis and non-cryptosporidiosis diarrhoea cases, in children under 2 years old. (PDF) [file pntd.0010508.s002.pdf]

Supplementary appendix for:

A comparison of risk factors for cryptosporidiosis and non-cryptosporidiosis diarrhoea: a hierarchical case-case-control study in Ethiopian children

CONTENTS

A selective review of the risk-factor literature for cryptosporidiosis 1

Supplementary methods 3

Recruitment of non-diarrhoea controls 3

Variables 4

Modelling strategy and statistical methods 5

Sample size considerations 5

Supplementary results 6

Table A: Distribution of case and control subjects according to all exposures, with counts and proportions of missing values 7

Table B: Hierarchical mediation analysis of risk factors for cryptosporidiosis and non-cryptosporidiosis diarrhoea, in children under 2 years old 8

Table C: Duration of diarrhoea, on enrolment, in cryptosporidiosis and non-cryptosporidiosis diarrhoea cases, in children under 2 years old 9

Bias analysis: Missing outcome for diarrhoea cases 10

Bias analysis: Enrolment of controls from vaccination rooms and by household visit 10

Bias analysis: Differential exposure misclassification for the sanitation variable 11

References 11

A selective review of the risk-factor literature for cryptosporidiosis

We have not performed a comprehensive or new systematic review of the diverse risk-factor literature for *Cryptosporidium* infection and cryptosporidiosis, but have chosen to use the most recent (2018), good-quality systematic review as a starting point [1]. We then summarize some key studies conducted thereafter, with a focus on the following: 1) how methodological challenges related to case ascertainment and confounding were managed, and 2) the relevance and applicability of the findings to clinical paediatric cryptosporidiosis in LMIC. As acute malnutrition was not included in the systematic review, we give a summary of a non-systematic review of the literature on acute malnutrition as a risk factor for both cryptosporidiosis and non-cryptosporidiosis diarrhoea (NCrD).

In summary, we identified evidence-gaps and unresolved discrepancies in the literature between risk factors for cryptosporidiosis and risk factors for pediatric diarrhoea in general [2, 3]. A recent meta-analysis identified animal contact, open defecation, and diarrheic household contacts as risk factors for *Cryptosporidium* infection; surprisingly, poor quality drinking water and lack of breastfeeding were not [1]. Later, the MAL-ED community-cohort study reported a limited set of risk factors for *Cryptosporidium* infection in children below 2 years of age[4]; only overcrowding and low length-for-age in the preceding months were found to be important risk factors, and, notably, unprotected drinking water, animal exposure, and sanitation factors were not. A recent study in the GEMS site in Gambia investigated *Cryptosporidium* infection in children presenting to healthcare with diarrhoea. Household water storage and the presence of cattle and cats in the household were reported as risk factors; overcrowding was, counterintuitively, found to be protective [5]. The frequent reports of animal exposure as a risk factor are contradicted by the currently available evidence from low-income settings [6]. The comparators used in most risk factor studies are diarrhoea cases without *Cryptosporidium* infection, i.e., case-case comparisons [7-14]. We have not identified any case-control studies that distinguished between *Cryptosporidium* infection and cryptosporidiosis, i.e., diarrhoea attributed to *Cryptosporidium* infection [15, 16], or that investigated hand hygiene, perinatal factors, and acute malnutrition in the same analysis [5, 17].

When trying to identify evidence gaps in the literature, the interpretation of many reports is complicated by the distinctions between exposure, infection, and disease frequently being unclear. To classify previous studies, we found it useful to consider separately each step that leads up to a case of cryptosporidiosis that presents for healthcare. We start by defining the population, e.g., all children under 5 who live in a geographically defined area around a hospital or health center. Within this "catchment area" different subsets of children will be exposed to different enteric microorganisms, including *Cryptosporidium*, and, among these subsets, some children will become infected. A proportion of those infected will develop diarrhoeal symptoms due the infection, i.e., cryptosporidiosis, and a subset of these will seek health care.

Interventions can, and should be, targeted at each step. Study designs and methods vary widely, and it follows that there will also be large variation in the applicability of risk-factor findings to the development of new interventions.

The 2018 systematic review on risk factors for *Cryptosporidium* infection in LMIC included only 15 studies and the authors highlighted the paucity of good-quality evidence [1]. The meta-analysis identified the following risk factors for infection (note; the review did not distinguish clearly between asymptomatic *Cryptosporidium* infection and cryptosporidiosis): animal contact, open defecation, and diarrhoea in a household contact. Surprisingly, poor quality drinking water and lack of breastfeeding were not risk factors [1]. It is worth noting that perinatal and previous illness factors were not included, hand hygiene was too infrequently reported for inclusion in the meta-analysis, none of the included studies investigated unsafe disposal of children's stools, and chronic or acute malnutrition were not reviewed as risk factors. As was also highlighted by the authors, particular care should be taken when interpreting the meta-analyses, as there was substantial heterogeneity between the 15 studies included, comprising both children and adults, asymptomatic and symptomatic infections, and studies conducted both within and outside a clinical setting. The authors pooled odds ratios (OR) from univariable analyses, precluding adjustment for important confounders. Seasonal effects and demographic and socioeconomic factors were not included yet can be important confounders of any inference about risk factors related to household transmission pathways, hygiene, nutritional status, or previous illness. Adjustment for socioeconomic, maternal, and perinatal factors is considered particularly important when quantifying the risk from early cessation of breastfeeding [18]. However, the meta-analysis of the protective effect of breastfeeding (figure 7 in [1]) pooled crude OR data from four studies that reported a protective effect, with one study reporting a non-significantly increased risk from breastfeeding. This latter study was conducted in an urban slum area with notably low rates of exclusive breastfeeding (<17%) and might represent an outlier [19].

A more recent publication on *Cryptosporidium* infection in children less than 2 years of age from the MAL-ED investigators included a multivariable analysis, with some adjustment for socioeconomic factors, but the set of included risk factors was limited [4]. Overcrowding and low preceding length-for-age were found to be important risk factors, and, notably, unprotected drinking water, animal exposure, and sanitation factors were not. It is worth noting that the authors did not differentiate between asymptomatic *Cryptosporidium* infection and cryptosporidiosis, and that the findings might not be applicable to clinical cryptosporidiosis as the study was conducted in a community setting.

Another recent study, from the GEMS site in Gambia, looked at *Cryptosporidium* infection in children presenting to healthcare with diarrhoea. Overcrowding was, counterintuitively, found to be protective, and household water storage and the presence of cows and cats in the household were reported as risk factors [5]. This study did not distinguish between diarrhoea-associated infection (i.e., cryptosporidiosis) and asymptomatic infection. The GEMS and MAL-ED "re-analysis" studies performed quantitative PCR testing and highlighted the important difference between *Cryptosporidium* infection and cryptosporidiosis, i.e., diarrhoea attributed to *Cryptosporidium* infection [15, 16], but none of the above studies applied a quantitative approach to the analysis of risk factors. Finally, the frequent reports of animal exposures as a risk factor are puzzling; *Cryptosporidium* is largely considered to have an anthroponotic transmission pattern in LMIC, and, in sub-Saharan Africa, a significant contribution from zoonotic infections is contradicted by the currently available evidence [6].

Most of the reviewed studies either reported crude associations or used multivariable logistic regression with stepwise elimination starting with all variables that were significant in initial models; to our knowledge no cryptosporidiosis risk-factor studies used a multivariate modelling approach based on a pre-defined causal framework for the relationships between the included variables (for example, using a hierarchical framework to guide the analysis, by directed acyclic graphs, or by other types of causal diagrams).

Acute malnutrition is well established as a risk factor for frequency, duration, severity, and case fatality of diarrhoeal episodes [20-24], but the increase in risk can be subtle unless the malnutrition is moderate-to-severe [23, 24]. For specific diarrhoeal aetiologies, the evidence is patchy, e.g., for cryptosporidiosis, none of the risk factor studies summarized above reported on moderate or severe acute malnutrition. However, several studies have compared characteristics in diarrhoea cases with and without *Cryptosporidium* infection (i.e., case-case comparisons) and observed an association between either being underweight (as measured by weight-for-age Z score, WAZ) wasting (as measured by weight-for-height Z score, WHZ) and/or severe acute malnutrition (SAM; defined by either severe wasting or bilateral oedema) and *Cryptosporidium* infection [7-14, 25]. Protein malnutrition, thought to be a key component in acute malnutrition, has been found to be a risk factor for cryptosporidiosis in animal models [26-28], however, the evidence from prospective studies in children is limited. A community birth cohort study conducted in Guinea-Bissau from 1987-1990 found no significant difference in absolute weight, WAZ, or length in children with cryptosporidiosis, compared with non-diarrhoea controls, measured at a time point within the preceding 90 day-period, instead reporting an association with subsequent weight loss (both short- and long-term) in the 0-180-day period after the episode [29, 30]. The study design did not allow disentangling whether this pattern was shared with NCrD or specific to cryptosporidiosis. A negative impact on ponderal growth after an episode of cryptosporidiosis is also supported by animal studies [31]. A birth cohort study from Peru found an association with (short-term) weight loss in the months after *Cryptosporidium* infection in a study that mainly included asymptomatic cases [32, 33]. Although no significant difference was seen in preceding weight between children with and without cryptosporidiosis, these two cohorts included few children with acute malnutrition, moderate-to-severe wasting, or severe underweight; the Peru cohort had three children with WHZ < -2; the Guinea-Bissau study did not specifically report the numbers with acute malnutrition (or WHZ) but noted little difference in mean WAZ preceding the episode (mean WAZ -0.74 in children with cryptosporidiosis, versus -0.76 in matched controls). Although not directly comparable, as conducted in 2-to-5-year-olds, a cohort study in a Bangladeshi slum reported that children who were underweight (WAZ was < -2 in 39% of the participants) had a significantly increased risk of subsequent cryptosporidiosis [34].

None of the cohort studies summarized above investigated moderate or severe acute malnutrition, as defined by WHZ or mid-upper-arm circumference (MUAC) cutoffs or the presence of oedema, as a risk factor for subsequent cryptosporidiosis incidence or severity. However, a recent post-hoc analysis of GEMS data investigated the interaction between acute malnutrition and healthcare-presenting diarrhoea and assessed the influence of acute malnutrition on diarrhoeal case-fatality risk at 60-day follow up [17]. This is likely the most comprehensive analysis of acute malnutrition as a risk factor for healthcare-presenting diarrhoea when broken down by aetiology. Furthermore, it stands out, as it compared cases to non-diarrhoea controls, accounted for confounding by age and key socioeconomic and water/sanitation/hygiene actors, and included both the severe and moderate form of acute malnutrition. The prevalence of *Cryptosporidium* (by ELISA antigen detection) in under-2-year-old diarrhoea cases was 17% in cases with acute malnutrition (vs 9% in controls) and 9% in cases without acute malnutrition (vs 4% in controls). Furthermore, acute malnutrition plus *Cryptosporidium* infection was associated with an additional increased absolute risk of death [17]. It is worth noting that quantitative cutoffs were not applied in the case ascertainment, and that only moderate-to-severe diarrhoea and diarrhoea of shorter than 7-days duration on presentation were included. To some extent, this limits the external applicability of their findings to the general paediatric population with diarrhoea. Furthermore, the authors did not present an analysis of other risk factors for *Cryptosporidium* infection, such as household water/sanitation factors or previous illness, to allow comparison of the strength of association or attributable fraction from various exposures. Lastly, although they adjusted for confounding by socioeconomic status and water/sanitation, as is sometimes also done in case-case-comparison studies, they may not have accounted for the possibility for separate confounding from behavioural hygienic factors or perinatal factors.

Supplementary methods

Recruitment of non-diarrhoea controls

Controls without diarrhoea in the preceding 48 hours were enrolled concurrently by weekly recruitment plans in two predefined geographical catchment areas comprising 15 municipal districts ("kebeles" - the smallest administrative unit in Ethiopia) surrounding JMC (Jimma Medical Center, formerly Jimma University Specialized Hospital), and eight districts surrounding Serbo Health Center (SHC). Plans regarding which controls to recruit were made every week using a method of frequency matching (i.e., by strata) to cases by geographical location of households, week, and age stratum (0-5 months, 6-11 months, 12-23 months, 23-59 months). Due to logistical constraints, controls in the age range 0-11 months were enrolled both from vaccination rooms in JMC and SHC and by home visits, whereas all controls in the age range 12-59 months were enrolled by home visits. Although frequency matching by both age, week, and geography was applied to all controls, irrespective of control

enrolment location, a separate analysis was conducted to assess for the possibility of enrolment-location selection bias (supplementary results, below). Nurses conducted random house visits in the community by car, motorcycle, and on foot. Randomization of households for control recruitment within each district was accomplished by a procedure where the nurse would start from a randomly selected GPS point within the district specified in the control recruitment plan, and then follow a detailed standardized procedure for household selection. For further details of the design and methods used in the CRYPTO-POC case-control sub-study, see two previous publications [35, 36]

Variables

Most variables are self-explanatory, with the following exceptions:

- *Number of key assets owned by the household.* A proxy for socioeconomic status based on the presence or absence of key household assets. We collected data on the same eight assets that were part of the composite asset index used in the MAL-ED study [37], except for household members/room (instead analysed as a separate risk factor).
- *Household sanitation facility*. The following facility types were considered as improved facilities: Flush toilet (WC) or pour flush toilet that flushed to a piped sewer system or septic tank or pit latrine; ventilated improved pit (VIP) latrine; pit latrine with slab; composting toilet. The following facility types were considered as unimproved facilities: Open pit or pit latrine without slab; bush, field, or no facility. Only improved sanitation that is not shared with other households was categorized as “access to improved sanitation”, according to the WHO definition.
- *Early cessation of exclusive breastfeeding*. Any cessation of exclusive breastfeeding earlier than the age of 6 months (the WHO minimum recommended age for weaning or for introducing complementary foods).
- *Acute malnutrition.* For children < 6 months, severe acute malnutrition (SAM) was defined as weight-for-height z-score (WHZ) ≤ -3 of the WHO standard curves [38] and/or presence of bilateral oedema involving at least the feet. Moderate acute malnutrition (MAM) was defined as a WHZ ≤ -2 and > -3 with no oedema. Mid-upper arm circumference (MUAC) was used instead of WHZ for 6-59-month-olds, as it was difficult to bring height measurement boards to community-control home visits (usually done by motorcycle), and because MUAC is less susceptible to dehydration than weight [39, 40]; SAM was defined as MUAC ≤ 115 mm and/or presence of bilateral oedema involving at least the feet, and MAM was defined a MUAC >115 mm and ≤ 125 mm with no oedema.

The case and control report forms also included questions about the following variables that were not included in the current analysis:

- *Household monthly income*. Intended to be used as an additional proxy for socioeconomic status, but not included in the analysis because income information was missing for >70% of the participants.
- *Handwashing hygiene*. Questions about the caregivers’ normal handwashing habits also included responses for caregiver handwashing following defecation of the child, and before breastfeeding. Due to high risk of differential recall bias, we did not use these variables in case-control comparisons.
- *Rotavirus vaccine, other vaccinations, and vitamin A administration.* Not deemed appropriate for case-control comparisons, as most of the infant controls were enrolled from vaccination rooms, with resulting risk of selection bias. Note that rotavirus vaccine had been introduced in the study area at the time of the study (94% of the study diarrhoea cases had received the vaccine [35]).
- *Stunting*. Not included in the analysis as height was not measured for most controls recruited in the community, as mentioned above.
- *Antibiotics, oral rehydration salts, or zinc treatment (received last week)*. Not deemed suitable for case-control risk exposure comparison, as we were not able to ascertain whether these treatments were given before or after the diarrhoeal episode started.
- *HIV status*. Not included due to infrequent exposure; the parent case-control study found only one HIV-exposed control, one HIV exposed NCrD case, and no HIV positives [35].
- *Immunosuppressive medical conditions*. Not included due to infrequent exposure; no participants reported other immunosuppressive conditions, e.g., lymphoma, leukaemia, other cancers, chronic kidney failure, or any condition requiring regular treatment with steroids.
- *Measles episode (during the last month)*. Not included due to infrequent exposure; only reported in three controls, and in two and three cases with cryptosporidiosis and NCrD, respectively.

Note that the reference levels used when estimating ORs for exposure-outcome associations are listed as a separate column in the result tables, for each variable.

Modelling strategy and statistical methods

We did not assume that the frequency-matching procedure was sufficient to account for confounding by age, study site, and season. Instead, adjustment was forced into all models by adding a fixed effect term for age in months, and by adding random-effect intercepts for enrolment season (divided into six three-month intervals) and study site to account for epidemiologic differences by site and season. Although gender was not part of the frequency-matching procedure, it was considered a likely confounder of all level-1, -2, -3, -4 and -5 risk-factor associations, and was therefore forced into all models.

Age, gender, study site, and season were considered as the base-adjustment set. Models, both with and without base adjustment, were compared to assess the impact of adjustment, if any, on the model estimates; if deemed necessary, we performed additional adjustment for sub-site geographical area, age stratum, or by smaller enrolment-time divisions.

The modelling strategy was an interactive step-by-step process starting with base-adjusted modes, followed by intra-level models, and finally, a hierarchical analysis accounting for all levels. Importantly, the final estimate for the overall effect of a distal variable is the estimate derived before the introduction of more proximal level risk factors.

First, ORs were estimated for all putative risk factors using only base-adjusted models. Any risk factor that was statistically significant at the 0.05 significance level (i.e., by the 95% confidence interval for the OR not overlapping 1) was then entered into an intra-level multivariable model, followed by backwards stepwise elimination until only significant risk factors (at the 0.05 significance level) remained.

Finally, significant risk factors from the intra-level models were introduced into the hierarchical risk factor analysis following the predefined order in the conceptual framework: first, starting with socioeconomic (level 1) variables that were significant in the level 1 intra-level analysis, we then proceeded to add risk factors from the level 2 intra-level model, followed by a new backwards stepwise elimination procedure. To the resulting new model, we then added the significant risk factors (if any) from the next level. A similar procedure was repeated for all hierarchical levels. Importantly, the final estimate for the overall effect of distal variable is the estimate derived before the introduction of more proximal risk factors levels.

We assessed whether associations between risk factors and disease followed a dose-response relationship by both exploratory plots and by separate models with continuous variables added as linear fixed-effect terms. For categorical variables with three or more levels, we performed separate models with added ordered linear and quadratic terms and used the P value to assess the statistical significance of the linearity of the association.

Co-linearity between the variables in multivariable models was estimated by the variance inflation factor (using the R package performance, v.0.5), and for continuous variables, by exploratory plots. All putative risk factors were also assessed for interaction with age by separate models by adding an interaction term for age group (<12 versus ≥ 12-months old), and by plots.

Population attributable fractions (PAF) were estimated with the formula $PAF = Prevalence\ x\ (1 - \frac{1}{OR})$, using the imputed prevalence of the risk factor in the case group and the OR estimate for the association between the risk factor and either disease [41]. The summary PAFs for the risk factors at a given level were derived from models that were adjusted for more distal levels, but not including more proximal levels [42]. A summary PAF for all levels was also calculated, by taking the complement of the PAF at each level. We also estimated the “non-mediated portion” of PAF at each level, i.e., the attributable fraction assumed to have resulted in disease by some other route than the intermediate risk factors explored, by calculating PAF after adding to the models any significant risk factors from more proximal levels.

Sample size considerations

The sample size for the CRYPTO-POC case-control substudy was determined as one of the primary objectives of the CRYPTO-POC diagnostic accuracy study, which required determining the association between *Cryptosporidium* detection in stool and diarrhoea (i.e., case/control status). For this objective, the aim was to reach

at least 90% power to detect a difference between cases and controls, assuming a prevalence of 1.5% in the control group, and an OR between cases and controls of at least 3, with a 95% two-sided confidence level. From this, a target minimum sample size of 728 cases and 728 controls was determined. Cryptosporidiosis risk-factor assessment (the current analysis) was a predefined objective of the CRYPTO-POC-study, but as it was a secondary objective, a separate sample size calculation was not performed. We assumed that the power would be lower, due to the smaller case set with cryptosporidiosis, but that the resulting reduction in power would be somewhat counteracted by an expected much higher than 1.5% prevalence for most of the exposures.

Supplementary results

Table A: Distribution of case and control subjects according to all exposures, with counts and proportions of missing values

Of all the modelled risk-factor variables, there was at least one missing observation for 5% of the cryptosporidiosis cases, 53% of the NCrD cases, and 49% of the controls. The highest proportion of missing values for any given exposure variable was 2.6% for controls, 3.4% for cryptosporidiosis cases, and 1.9% for NCrD cases. We therefore performed multiple chained random-forest imputation before all OR and PAF calculations.

				Diarrhoea cases					
	Non-diarrhoea controls (N = 725)			Cryptosporidiosis (N = 59)			Non-cryptosporidiosis (N = 432)		
Characteristic	Exposed n	Missing n	Missing %	Exposed n	Missing n	Missing %	Exposed n	Missing n	Missing %
**Level 1 – Socioeconomic factors**									
Maternal education		2	0.3		0	0.0		1	0.2
< 1 year	198			21			150		
1–7 years	273			19			129		
Primary caregiver is not the child's mother	13	2	0.3	6	0	0.0	25	0	0.0
Number of key assets owned by the household ≤ 2	115	14	1.9	8	2	3.4	103	6	1.4
Number of household members		9	1.2		1	1.7		2	0.5
4-5	336			28			170		
≥ 6	199			13			156		
**Level 2 – Household environmental factors**									
Persons per room ≥ 2	675	3	0.4	51	0	0.0	390	2	0.5
Animals owned by the household (household ownership of ≥1 animal of the stated type)		2	0.3		0	0.0		1	0.2
Cattle	244			16			180		
Chickens	233			16			145		
Dogs	62			8			30		
Goats	54			4			41		
Horses, donkeys, or mules	66			4			54		
Sheep	107			6			61		
Other	23			1			6		
Any even-toed ungulate	260			19			184		
Any animal	357			27			217		
Sanitation facility		0	0.0		0	0.0		1	0.2
Improved, but shared	62			7			82		
Improved, and not shared	59			22			124		
Access to "improved sanitation" (by the WHO definition)	59	0	0.0	22	0	0.0	124	1	0.2
Water source for the household		2	0.3		0	0.0		1	0.2
Public tap	98			22			106		
Surface or rainwater, unprotected well, borehole, or protected spring	221			9			154		
Water treated by the household (chemicals, boiling or filtering) before drinking	38	4	0.6	3	0	0.0	41	5	1.2
**Level 3 – Hygiene behaviour**									
Last stool disposal (from any child of the caregiver) "unsafe" by the WHO definition	404	1	0.1	39	0	0.0	327	1	0.2
Caregiver will normally wash hands									
before meals	686	0	0.0	55	0	0.0	398	0	0.0

before preparing food for the child	538	0	0.0	46	0	0.0	315	0	0.0
after a toilet visit	497	0	0.0	38	0	0.0	303	0	0.0
without soap	24	0	0.0	3	0	0.0	30	0	0.0
**Level 4 – Perinatal factors**									
Mode of delivery – caesarean section	47	1	0.1	7	1	1	47	1	0.2
Child born prematurely (before week 37)	14	0	0.0	3	1	1.7	25	2	0.5
**Level 5 – Breastfeeding, nutritional status, and previous illness history**									
Early cessation of exclusive breastfeeding	231	4	0.5	19	0	0.0	164	8	1.9
Not breastfeeding now (or, for cases, just before the diarrhoeal episode started)	61	1	0.1	9	0	0.0	48	0	0.0
Acute malnutrition		19	2.6		0	0.0		5	1.2
Moderate acute malnutrition (MAM)	16			7			45		
Severe acute malnutrition (SAM)	4			4			9		
Acute malnutrition, any (MAM or SAM)	20			11			54		
One or more overnight admissions, since birth	53	1	0.1	6	0	0.0	34	2	0.5
One or more diarrhoea episodes, during the last month	109	0	0.0	16	0	0.0	73	4	0.9
Number of visits to hospital or health center due to illness, since birth		0	0.0		0	0.0		0	0.0
1-2 visits	194			21			126		
≥ 3 visits	3			7			16		

Table B: Hierarchical mediation analysis of risk factors for cryptosporidiosis and non-cryptosporidiosis diarrhoea, in children under 2 years old

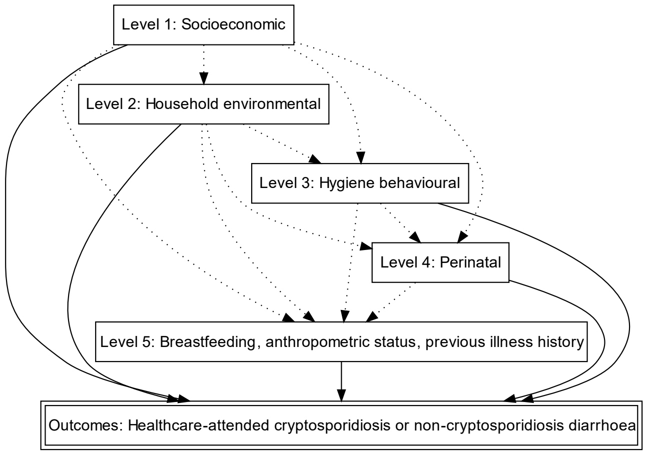

The conceptual framework illustrates our underlying assumption that a risk factor can have a causal relationship with disease via several routes. In this version of the conceptual framework (figure, left), "mediated" routes are marked by dotted lines to distinguish them from the "non-mediated" routes (whole lines).

A mediation analysis is possible by deliberately adjusting for a mediator, i.e., a proximal and intermediate risk factor in the causal framework. The approach taken here is a simple variant of the traditional "difference method" of quantifying mediation [43], which is based on comparing regression coefficients (or, in logistic regression models, odds ratios) between two models, where one includes adjustment for a mediator, and the other does not include such adjustment. Odds ratios (ORs), and PAFs based on ORs, can then be estimated both with and without this adjustment. Mediator adjustment should only be done as part of a mediation analysis, as it can lead to underestimation of ORs (and population attributable fractions, PAFs) for distal risk factors (i.e., a type of overadjustment). Mediation analysis presupposes certain causal relationships, usually illustrated by directed acyclic graphs or a hierarchical framework, as above. Under the underlying assumptions implicit in the framework, a significant drop in OR, after adjusting for a proximate and intermediate step (i.e., a "mediator") provides indirect evidence for mediation of the distal risk factor through this intermediate step. As PAFs are calculated from ORs, this approach allows us to split a PAF (either a PAF from a specific risk factor or a whole-level summary PAF) into "non-mediated" and "mediated" fractions. The term "non-mediated" in this context should be cautiously interpreted, i.e., "not likely to be mediated by the other risk factors we gathered data on".

This table shows how the intra-level summary PAFs change as mediator adjustment for more proximal levels are added to the models:

Level	Cryptosporidiosis vs controls				Non-cryptosporidiosis diarrhoea vs controls			
	Summary PAF from each level (%)[a]	Non-mediated PAF (%)[b]	Mediated PAF (%)	Mediated vs non-mediated fraction of PAF (%)	Summary PAF from each level (%)[a]	Non-mediated PAF (%)[b]	Mediated PAF (%)	Mediated vs non-mediated fraction of PAF (%)
Level 1 – Socioeconomic factors	26	8	18[c]	68	14	11	3	22
Level 2 – Household environmental factors	27	27	0	0	31	30	1	1
Level 3 – Hygiene factors	0				44	40	4	8
Level 4 – Perinatal factors	0				8	8	0	0
Level 5 – Breastfeeding, nutrition, previous illness	39	39	0	0	22	22	0	0

[a] Summary PAFs were calculated for each risk factor level by taking the complement of PAFs for individual significant risk factors at that level. The summary PAFs for the risk factors a given level were derived from models that were adjusted for more distal levels, but not including more proximal levels.

[b] "Non-mediated PAF" is calculated from odds ratios obtained from models with fixed effect terms for more distal risk factors (i.e., confounder adjustment), and also terms for more proximal risk factors (i.e., mediator adjustment). This "non-mediated portion of PAF" is an estimate of the attributable fraction assumed to have resulted in disease by some other route than the intermediate risk factors explored, by calculating PAF after adding to the models any significant risk factors from more proximal levels.

[c] Maternal education <1 year becomes insignificant (with a resulting drop in PAF), after mediator adjustment for level 2, indicating mediation of this risk factor through level 2 risk factors.

Table C: Duration of diarrhoea, on enrolment, in cryptosporidiosis and non-cryptosporidiosis diarrhoea cases, in children under 2 years old

	Cryptosporidiosis (N = 59)[a]	Non-cryptosporidiosis diarrhoea (N = 432)[a]
Diarrhoeal duration, days; median (IQR)	3 (3-7)	3 (2-4)
Diarrhoeal duration, days; mean (SD)	4.6 (2.9)	4 (5.4)
Acute diarrhoea (2-6 days duration)	42 (71)	375 (87)
Prolonged diarrhoea (7-13 days duration)	14 (24)	43 (10)
Persistent diarrhoea (14 days duration or longer)	3 (5)	14 (3)

IQR = interquartile range. SD = standard deviation.

[a] Data are n (%), unless otherwise specified.

Bias analysis: Missing outcome for diarrhoea cases

The primary outcome of either cryptosporidiosis or non-cryptosporidiosis diarrhoea depended on successfully obtaining a stool sample for reference testing. Failure to obtain samples for all (logistically challenging as most cases were outpatients), and a few missing reference tests (see flowchart in Figure 1, main manuscript), meant that outcome status was not known for all participants. If missing outcomes are associated with risk factors under investigation, it can lead to selection bias. To detect such associations between missing outcome and exposures, all diarrhoea cases in the study were enrolled, interviewed, and examined, irrespective of the availability of a stool sample or of reference testing. We then explored differences in exposures between diarrhoea cases, with and without missing outcomes, by plots and cross tabulation, for all risk factor variables.

For most of the investigated variables, including the frequency-matching variables (age, enrolment season, study site, geographical area), there were no substantial exposure differences by missing-outcome-status, and therefore low suspicion of selection bias. However, for the following variables, we observed a small yet substantial difference: gender (higher proportion of girls among those with missing outcome), maternal education (1-7 years reported more often for cases with missing outcome), water source (unimproved water source reported more often and public tap less often among cases with missing outcome), and history of diarrhoea (reported less often by cases with missing outcome). We calculated the selection bias factor and selection bias-adjusted crude odds ratios [44], under the assumption that risk of missing outcome (i.e., probability of not being selected for analysis), was similar between cryptosporidiosis and non-cryptosporidiosis cases. See table below for crude and adjusted ORs. The results indicate that the ORs for public tap water and previous history of diarrhoea may be biased slightly upwards, due to missing-outcome selection bias.

		**Cryptosporidiosis**		**Non-cryptosporidiosis diarrhoea**	
**Characteristic**	**Selection bias factor**[a]	**Crude OR**[b]	**Adjusted OR**[c]	**Crude OR**[b]	**Adjusted OR**[c]
Male gender	1.13	0.76 (0.45 to 1.3)	0.67 (0.39 to 1.2)	1.3 (1.0 to 1.7)	1.1 (0.89 to 1.5)
Maternal education					
≥ 8 years	(Reference level)				
< 1 year	1.04	1.4 (0.74 to 2.7)	1.4 (0.69 to 2.6)	1.1 (0.80 to 1.5)	1.0 (0.77 to 1.4)
1–7 years	1.23	0.92 (0.48 to 1.8)	0.75 (0.37 to 1.4)	0.91 (0.69 to 1.2)	0.74 (0.55 to 0.99)
Water source for the household					
Private tap	(Reference level)				
Public tap	1.18	3.2 (1.8 to 5.9)	2.8 (1.5 to 5.1)	2.6 (1.8 to 3.6)	2.2 (1.6 to 3.0)
Surface or rainwater, unprotected well, borehole, or protected spring	0.94	0.59 (0.3 to 1.3)	0.63 (0.28 to 1.5)	1.7 (1.3 to 2.2)	1.8 (1.3 to 2.3)
One or more diarrhoea episodes, during the last month	1.22	2.1 (1.1 to 3.9)	1.7 (0.93 to 3.4)	1.2 (0.84 to 1.6)	0.95 (0.68 to 1.33)

OR = odds ratio.

[a] Selection bias factor = $S_{Aj}S_{B0} / S_{A0}S_{Bj}$, where S_{Aj} is the probability of selecting an exposed case, S_{A0} is the probability of selecting an unexposed case, S_{Bj} is the probability of selecting an exposed control, and S_{B0} is the probability of selecting an unexposed control.

[b] Point OR estimates with 95% Wald confidence intervals.

[c] Selection bias factor corrected OR, with 95% confidence intervals obtained using bootstrapping with 100 replicates, using the R package episensr (v.1.0.0).

Table D: Selection bias adjusted crude odds ratios, under 2-year-olds

Bias analysis: Enrolment of controls from vaccination rooms and by household visit

Most controls in the age group 0-11-months were enrolled from vaccination rooms in JMC and SHC, due to logistical constraints on conducting field visits. If we were unsuccessful in recruiting a control from the matching age stratum (0-5-months; 6-11 months) or geographical area, a similar control (by age stratum and geographical area) was enrolled the following week, but by the procedure for random house visits. Only 12% of the 0-11-month-old controls (50/414) were enrolled by house visits. House visit enrolment, instead of vaccination room enrolment, was mainly associated with geographical distance from the household to the healthcare facility. In

contrast, all controls ≥ 12 months old were enrolled by house visits. We therefore considered selection bias, by control recruitment location, as a real possibility, for all putative risk factors, despite geography being largely accounted for by the frequency matching procedure, and despite close harmonization of study procedures irrespective of recruitment strategy.

For this kind of selection bias to have an important effect on the exposure-outcome associations (i.e., ORs), we would expect to see substantial differences in OR by age group (below or above 12 months old; all controls ≥ 12 months were enrolled by house visits), and, also, evidence of substantial differences in exposure by control recruitment location. We also used information on the approximate distance from the household to the health facility, and the caregiver-stated preferred facility for childhood vaccinations, from both cases and controls (e.g., if the average case preferred to get vaccinated elsewhere than the average control, it was considered evidence against the assumption that they were drawn from the same underlying source population, or "study base"). Based on this, we took the following systematic approach to examine for control selection bias: for all base adjusted exposure-outcome relationships, we assessed OR differences by age group, and for substantial difference in exposure prevalence between home-recruited and vaccination-recruited controls (by inspecting the numbers, or by finding a substantial difference in exposure prevalence, as estimated by 95% Wilson confidence intervals). If present, we then proceeded to run the models both with and without adjustment and, separately, with interaction terms for vaccination location preference and by geographical distance of the household to JMC/SHC. If the OR for the exposure-outcome relationship changed substantially, it was considered evidence of control selection bias. If not, such bias was considered less likely.

Using this approach, we discovered that for the association between cessation of exclusive breastfeeding before the age of 6 months and NCrD, there was both an association with age-group (increased risk of NCrD, but not cryptosporidiosis, in the 0-11-month-olds) and a difference in exposure based on control recruitment location (22% of the vaccination room controls had stopped being exclusively breastfed earlier than 6 months versus 40% of the household controls). However, as there was minimal change in OR after adjusting for geographical distance to the health facility or when adjusting for vaccination facility preference, selection bias was not considered likely. No evidence for control selection bias was found for any of the other putative risk factors.

Bias analysis: Differential exposure misclassification for the sanitation variable

Somewhat surprisingly, the rate of access to improved sanitation was much higher in both cryptosporidiosis cases and NCrD cases than in the control group (Table 2, main manuscript). We wondered whether the complexity of how to define "improved facility", could lead to misclassification based on the individual study nurse conducting the questionnaire interview. Some misclassifications are to be expected in a large study. However, only if such misclassifications occur often, and are differential, i.e., if the misclassification operates differently for participants based on outcome (e.g., case-control status), is it likely to lead to bias. As the proportion of case to control recruitment varied to some extent by study nurse, we could not rule out differential misclassification as a possibility. In order not to single out the sanitation variable for special scrutiny, we therefore proceeded to investigate all modelled exposure variables for possible differential exposure misclassification by study nurse. The only variable with evidence of differential clustering by study nurse was the sanitation variable. To take this clustering effect into account, we therefore added a random effect intercept for study nurse to all models that included the sanitation variable.

References

1. Bouzid M, Kintz E, Hunter PR. Risk factors for Cryptosporidium infection in low and middle income countries: A systematic review and meta-analysis. PLoS Negl Trop Dis. 2018;12(6):e0006553. Epub 2018/06/08. doi: 10.1371/journal.pntd.0006553. PubMed PMID: 29879110; PubMed Central PMCID: PMCPMC6014672.
2. Troeger C.E., Khalil I.A., Blacker B.F., Biehl M.H., Albertson S.B., Zimsen S.R.M., et al. Quantifying risks and interventions that have affected the burden of diarrhoea among children younger than 5 years: an analysis of the Global Burden of Disease Study 2017. Lancet Infect Dis. 2020;20(1):37-59. Epub 2019/11/05. doi: 10.1016/s1473-3099(19)30401-3. PubMed PMID: 31678029; PubMed Central PMCID: PMCPMC7340495.
3. Bhutta ZA, Das JK, Walker N, Rizvi A, Campbell H, Rudan I, et al. Interventions to address deaths from childhood pneumonia and diarrhoea equitably: what works and at what cost? Lancet. 2013;381(9875):1417-29. Epub 2013/04/16. doi: 10.1016/s0140-6736(13)60648-0. PubMed PMID: 23582723.
4. Korpe PS, Valencia C, Haque R, Mahfuz M, McGrath M, Houpt E, et al. Epidemiology and Risk Factors for Cryptosporidiosis in Children From 8 Low-income Sites: Results From the MAL-ED Study. Clin Infect Dis.

2018;67(11):1660-9. Epub 2018/04/28. doi: 10.1093/cid/ciy355. PubMed PMID: 29701852; PubMed Central PMCID: PMCPMC6233690.
5. Hossain MJ, Saha D, Antonio M, Nasrin D, Blackwelder WC, Ikumapayi UN, et al. Cryptosporidium infection in rural Gambian children: Epidemiology and risk factors. PLoS Negl Trop Dis. 2019;13(7):e0007607. Epub 2019/07/28. doi: 10.1371/journal.pntd.0007607. PubMed PMID: 31348795; PubMed Central PMCID: PMCPMC6685629 Economics at GlaxoSmithKline (GSK) Vaccines, Wavre, Belgium. RA is retired from GSK and RA was a Global Director, Scientific Affairs of GSK Vaccines, Wavre, Belgium. Both of them were with MRC Unit The Gambia at LSHTM during the study period. The current work of DS does not involve research on Cryptosporidium vaccine. The other authors have declared that no competing interests exist.
6. Robertson LJ, Johansen ØH, Kifleyohannes T, Efunshile AM, Terefe G. Cryptosporidium Infections in Africa—How Important Is Zoonotic Transmission? A Review of the Evidence. Frontiers in Veterinary Science. 2020;7(724). doi: 10.3389/fvets.2020.575881.
7. Tumwine JK, Kekitiinwa A, Nabukeera N, Akiyoshi DE, Rich SM, Widmer G, et al. Cryptosporidium parvum in children with diarrhea in Mulago Hospital, Kampala, Uganda. Am J Trop Med Hyg. 2003;68(6):710-5. Epub 2003/07/31. PubMed PMID: 12887032.
8. Cegielski JP, Ortega YR, McKee S, Madden JF, Gaido L, Schwartz DA, et al. Cryptosporidium, enterocytozoon, and cyclospora infections in pediatric and adult patients with diarrhea in Tanzania. Clin Infect Dis. 1999;28(2):314-21. Epub 1999/03/04. doi: 10.1086/515131. PubMed PMID: 10064250.
9. Adjei AA, Armah H, Rodrigues O, Renner L, Borketey P, Ayeh-Kumi P, et al. Cryptosporidium Spp., a frequent cause of diarrhea among children at the Korle-Bu Teaching Hospital, Accra, Ghana. Jpn J Infect Dis. 2004;57(5):216-9. Epub 2004/10/28. PubMed PMID: 15507781.
10. Gay-Andrieu E, Adehossi E, Illa H, Garba Ben A, Kourna H, Boureima H. [Prevalence of cryptosporidiosis in pediatric hospital patients in Niamey, Niger]. Bull Soc Pathol Exot. 2007;100(3):193-6. Epub 2007/09/11. PubMed PMID: 17824314.
11. Duong TH, Dufillot D, Koko J, Nze-Eyo'o R, Thuilliez V, Richard-Lenoble D, et al. [Digestive cryptosporidiosis in young children in an urban area in Gabon]. Sante. 1995;5(3):185-8. Epub 1995/05/01. PubMed PMID: 7543796.
12. Javier Enriquez F, Avila CR, Ignacio Santos J, Tanaka-Kido J, Vallejo O, Sterling CR. Cryptosporidium infections in Mexican children: clinical, nutritional, enteropathogenic, and diagnostic evaluations. Am J Trop Med Hyg. 1997;56(3):254-7. Epub 1997/03/01. doi: 10.4269/ajtmh.1997.56.254. PubMed PMID: 9129526.
13. Delahoy MJ, Omore R, Ayers TL, Schilling KA, Blackstock AJ, Ochieng JB, et al. Clinical, environmental, and behavioral characteristics associated with Cryptosporidium infection among children with moderate-to-severe diarrhea in rural western Kenya, 2008-2012: The Global Enteric Multicenter Study (GEMS). PLoS Negl Trop Dis. 2018;12(7):e0006640. Epub 2018/07/13. doi: 10.1371/journal.pntd.0006640. PubMed PMID: 30001340; PubMed Central PMCID: PMCPMC6057667.
14. Deichsel EL, Hillesland HK, Gilchrist CA, Naulikha JM, McGrath CJ, Van Voorhis WC, et al. Prevalence and Correlates of Cryptosporidium Infections in Kenyan Children With Diarrhea and Their Primary Caregivers. Open Forum Infect Dis. 2020;7(12):ofaa533. Epub 2020/12/19. doi: 10.1093/ofid/ofaa533. PubMed PMID: 33335937; PubMed Central PMCID: PMCPMC7731525.
15. Liu J, Platts-Mills JA, Juma J, Kabir F, Nkeze J, Okoi C, et al. Use of quantitative molecular diagnostic methods to identify causes of diarrhoea in children: a reanalysis of the GEMS case-control study. Lancet. 2016;388(10051):1291-301. Epub 2016/09/28. doi: 10.1016/s0140-6736(16)31529-x. PubMed PMID: 27673470; PubMed Central PMCID: PMCPMC5471845.
16. Platts-Mills JA, Liu J, Rogawski ET, Kabir F, Lertsethtakarn P, Siguas M, et al. Use of quantitative molecular diagnostic methods to assess the aetiology, burden, and clinical characteristics of diarrhoea in children in low-resource settings: a reanalysis of the MAL-ED cohort study. Lancet Glob Health. 2018;6(12):e1309-e18. Epub 2018/10/06. doi: 10.1016/s2214-109x(18)30349-8. PubMed PMID: 30287127; PubMed Central PMCID: PMCPMC6227251.
17. Tickell KD, Sharmin R, Deichsel EL, Lamberti LM, Walson JL, Faruque ASG, et al. The effect of acute malnutrition on enteric pathogens, moderate-to-severe diarrhoea, and associated mortality in the Global Enteric Multicenter Study cohort: a post-hoc analysis. Lancet Glob Health. 2020;8(2):e215-e24. Epub 2020/01/26. doi: 10.1016/s2214-109x(19)30498-x. PubMed PMID: 31981554; PubMed Central PMCID: PMCPMC7025322.
18. Horta BL, Victora CG, WHO. Short-term effects of breastfeeding: a systematic review on the benefits of breastfeeding on diarrhoea and pneumonia mortality. Geneva: World Health Organization; 2013 2013.
19. Sarkar R, Kattula D, Francis MR, Ajjampur SS, Prabakaran AD, Jayavelu N, et al. Risk factors for cryptosporidiosis among children in a semi urban slum in southern India: a nested case-control study. Am J Trop Med Hyg. 2014;91(6):1128-37. Epub 2014/10/22. doi: 10.4269/ajtmh.14-0304. PubMed PMID: 25331810; PubMed Central PMCID: PMCPMC4257634.
20. Brown KH. Diarrhea and malnutrition. J Nutr. 2003;133(1):328s-32s. Epub 2003/01/07. doi: 10.1093/jn/133.1.328S. PubMed PMID: 12514320.

21. Guerrant RL, Schorling JB, McAuliffe JF, de Souza MA. Diarrhea as a cause and an effect of malnutrition: diarrhea prevents catch-up growth and malnutrition increases diarrhea frequency and duration. Am J Trop Med Hyg. 1992;47(1 Pt 2):28-35. Epub 1992/07/01. doi: 10.4269/ajtmh.1992.47.28. PubMed PMID: 1632474.
22. James JW. Longitudinal study of the morbidity of diarrheal and respiratory infections in malnourished children. Am J Clin Nutr. 1972;25(7):690-4. Epub 1972/07/01. doi: 10.1093/ajcn/25.7.690. PubMed PMID: 4624650.
23. Sepúlveda J, Willett W, Muñoz A. Malnutrition and diarrhea. A longitudinal study among urban Mexican children. Am J Epidemiol. 1988;127(2):365-76. Epub 1988/02/01. doi: 10.1093/oxfordjournals.aje.a114810. PubMed PMID: 3337088.
24. Schorling JB, McAuliffe JF, de Souza MA, Guerrant RL. Malnutrition is associated with increased diarrhoea incidence and duration among children in an urban Brazilian slum. Int J Epidemiol. 1990;19(3):728-35. Epub 1990/09/01. doi: 10.1093/ije/19.3.728. PubMed PMID: 2262271.
25. Kirkpatrick BD, Daniels MM, Jean SS, Pape JW, Karp C, Littenberg B, et al. Cryptosporidiosis stimulates an inflammatory intestinal response in malnourished Haitian children. J Infect Dis. 2002;186(1):94-101. Epub 2002/06/29. doi: 10.1086/341296. PubMed PMID: 12089667.
26. Costa LB, Noronha FJ, Roche JK, Sevilleja JE, Warren CA, Oriá R, et al. Novel in vitro and in vivo models and potential new therapeutics to break the vicious cycle of Cryptosporidium infection and malnutrition. J Infect Dis. 2012;205(9):1464-71. Epub 2012/03/29. doi: 10.1093/infdis/jis216. PubMed PMID: 22454464; PubMed Central PMCID: PMCPMC3324401.
27. Guerrant RL, Bolick DT, Swann JR. Modeling Enteropathy or Diarrhea with the Top Bacterial and Protozoal Pathogens: Differential Determinants of Outcomes. ACS Infect Dis. 2021. Epub 2021/04/27. doi: 10.1021/acsinfecdis.0c00831. PubMed PMID: 33901398.
28. Liu J, Bolick DT, Kolling GL, Fu Z, Guerrant RL. Protein Malnutrition Impairs Intestinal Epithelial Cell Turnover, a Potential Mechanism of Increased Cryptosporidiosis in a Murine Model. Infect Immun. 2016;84(12):3542-9. Epub 2016/11/01. doi: 10.1128/iai.00705-16. PubMed PMID: 27736783; PubMed Central PMCID: PMCPMC5116730.
29. Mølbak K, Andersen M, Aaby P, Højlyng N, Jakobsen M, Sodemann M, et al. Cryptosporidium infection in infancy as a cause of malnutrition: a community study from Guinea-Bissau, west Africa. Am J Clin Nutr. 1997;65(1):149-52.
30. Mølbak K, Højlyng N, Gottschau A, Sa JC, Ingholt L, da Silva AP, et al. Cryptosporidiosis in infancy and childhood mortality in Guinea Bissau, west Africa. BMJ. 1993;307(6901):417-20. Epub 1993/08/14. PubMed PMID: 8374453; PubMed Central PMCID: PMCPmc1678388.
31. Coutinho BP, Oriá RB, Vieira CM, Sevilleja JE, Warren CA, Maciel JG, et al. Cryptosporidium infection causes undernutrition and, conversely, weanling undernutrition intensifies infection. J Parasitol. 2008;94(6):1225-32. Epub 2008/06/26. doi: 10.1645/ge-1411.1. PubMed PMID: 18576767; PubMed Central PMCID: PMCPMC3070954.
32. Checkley W, Gilman RH, Epstein LD, Suarez M, Diaz JF, Cabrera L, et al. Asymptomatic and symptomatic cryptosporidiosis: their acute effect on weight gain in Peruvian children. Am J Epidemiol. 1997;145(2):156-63. Epub 1997/01/15. doi: 10.1093/oxfordjournals.aje.a009086. PubMed PMID: 9006312.
33. Checkley W, Epstein LD, Gilman RH, Black RE, Cabrera L, Sterling CR. Effects of Cryptosporidium parvum infection in Peruvian children: growth faltering and subsequent catch-up growth. Am J Epidemiol. 1998;148(5):497-506. Epub 1998/09/16. PubMed PMID: 9737562.
34. Mondal D, Haque R, Sack RB, Kirkpatrick BD, Petri WA, Jr. Attribution of malnutrition to cause-specific diarrheal illness: evidence from a prospective study of preschool children in Mirpur, Dhaka, Bangladesh. Am J Trop Med Hyg. 2009;80(5):824-6. Epub 2009/05/02. PubMed PMID: 19407131; PubMed Central PMCID: PMCPMC3410540.
35. Johansen ØH, Abdissa A, Zangenberg M, Mekonnen Z, Eshetu B, Bjørang O, et al. Performance and operational feasibility of two diagnostic tests for cryptosporidiosis in children (CRYPTO-POC): a clinical, prospective, diagnostic accuracy study. The Lancet Infectious Diseases. 2020. doi: https://doi.org/10.1016/S1473-3099(20)30556-9.
36. Zangenberg M, Johansen ØH, Abdissa A, Eshetu B, Kurtzhals JAL, Friis H, et al. Prolonged and persistent diarrhoea is not restricted to children with acute malnutrition: an observational study in Ethiopia. Trop Med Int Health. 2019;24(9):1088-97. Epub 2019/07/22. doi: 10.1111/tmi.13291. PubMed PMID: 31325406.
37. Berendes D, Leon J, Kirby A, Clennon J, Raj S, Yakubu H, et al. Household sanitation is associated with lower risk of bacterial and protozoal enteric infections, but not viral infections and diarrhoea, in a cohort study in a low-income urban neighbourhood in Vellore, India. Trop Med Int Health. 2017;22(9):1119-29. Epub 2017/06/28. doi: 10.1111/tmi.12915. PubMed PMID: 28653489; PubMed Central PMCID: PMCPMC5601219.

38. WHO Multicentre Growth Reference Study Group. WHO Child Growth Standards: Length/height-for-age, weight-for-age, weight-for-length, weight-for-height and body mass index-for-age: Methods and development. Geneva: World Health Organization (312 pages). 2006.
39. Mwangome MK, Fegan G, Prentice AM, Berkley JA. Are diagnostic criteria for acute malnutrition affected by hydration status in hospitalized children? A repeated measures study. Nutr J. 2011;10:92. Epub 2011/09/14. doi: 10.1186/1475-2891-10-92. PubMed PMID: 21910909; PubMed Central PMCID: PMCPMC3180351.
40. Modi P, Nasrin S, Hawes M, Glavis-Bloom J, Alam NH, Hossain MI, et al. Midupper Arm Circumference Outperforms Weight-Based Measures of Nutritional Status in Children with Diarrhea. J Nutr. 2015;145(7):1582-7. Epub 2015/05/15. doi: 10.3945/jn.114.209718. PubMed PMID: 25972523; PubMed Central PMCID: PMCPMC4478950.
41. Lin CK, Chen ST. Estimation and application of population attributable fraction in ecological studies. Environ Health. 2019;18(1):52. Epub 2019/06/15. doi: 10.1186/s12940-019-0492-4. PubMed PMID: 31196209; PubMed Central PMCID: PMCPMC6567453.
42. Ferrer SR, Strina A, Jesus SR, Ribeiro HC, Cairncross S, Rodrigues LC, et al. A hierarchical model for studying risk factors for childhood diarrhoea: a case-control study in a middle-income country. Int J Epidemiol. 2008;37(4):805-15. Epub 2008/06/03. doi: 10.1093/ije/dyn093. PubMed PMID: 18515864.
43. VanderWeele TJ. Mediation Analysis: A Practitioner's Guide. Annu Rev Public Health. 2016;37:17-32. Epub 2015/12/15. doi: 10.1146/annurev-publhealth-032315-021402. PubMed PMID: 26653405.
44. Lash TL, VanderWeele TJ, Haneause S, Rothman K. Modern epidemiology. 4th ed: Lippincott Williams & Wilkins; 2020. p. 729-30.
