# Supplementary material for: A comparison of risk factors for cryptosporidiosis and non-cryptosporidiosis diarrhoea: A case-case-control study in Ethiopian children
Source: PLoS Negl Trop Dis. 2022 Jun 6;16(6):e0010508. doi: 10.1371/journal.pntd.0010508 (PMC9203008; doi:10.1371/journal.pntd.0010508)
Supplement: Attachment — Submitted filename: PNTD 2021_07.docx [file pntd.0010508.s004.docx]

The author’s present a thoughtful, well written, and thorough analysis of risk factors for cryptosporidium and diarrhea in a population of children under two in Ethiopia. The manuscript applies a novel analytic strategy to covers an important and relevant public health topic. Overall, the manuscript would benefit from focus on the overall aim and additional information to the methods section.

Overall, I have never seen the case-case-control study design applied previously. I have some concerns that comparing the effect estimates from the two models are due the differences in sample size between the two models. A biostatistical should also review this paper with this specific method in mind.

More detailed suggestions are listed below:

Introduction:

1. The introduction could be shortened overall. Paragraph 2 does not need to include detailed conclusions about specific studies. This could be shorted to just stay the results for risk factors are inconsistent.
2. Paragraph 3 may be better included in the methods section. A shorter description of the aims and not the rational for comparison.
3. There are a lot of comparisons in this manuscript. It is unclear if one of the main goals is to compare the epidemiologic methods here (there are 3 sets of results presented in this paper making it challenging to understand the overall conclusion). If this is the goal, I suggest limiting the comparison between crypto and non-crypto diarrhea (as mentioned above). Focusing the manuscript will help with the interpretation. If the authors want to stick with crypto vs non diarrhea comparted to non-crypto diarrhea vs non-diarrhea, I suggest removing one of the interim analyses-table 3 for example. If you believe the multilevel models are the most appropriate way to analyses these data, then why also present a mis-specified model without these levels. Choosing the primary comparison to communicate will also help focus the introduction.
4. Line 90: I suggest specifying the comparison in case control as you did for case to case comparison. Add the comparison “diarrhoea attributable to cryptosporidium infection compared to children with no diarrhea”

Methods:

5. Please specify definition of diarrhea used. Any loose stools? ≥ 3 loose stools in 24 hrs?
6. Please specify how you defined non-diarrheal controls. No diarrhea at time of selection? No diarrhea in past 7 days? 14 days?
7. Please specify if the non-diarrhea children had any stool testing done. Some of these may have asymptomatic infections.
8. Please specify how the composite was used to define crypto positive. Was a child with a positive crypto result by any method considered positive? Did it need to be 2 out of 3 tests to be crypto positive?
9. Please list CT cut off values used in the methods section so the reader doesn’t have to access the reference.
10. I disagree that all variable definitions are self explanatory. Please include a brief definition of malnutrition and the cut off values in the methods section. I suggest rewording this sentence to say *other* definitions can be found in the appendix.
11. Please include some information how the list of risk factors were determined in the manuscript, more detail can be kept in the supplemental materials. You could just say the list of risk factors was largely taken from key published papers. The source of perinatal factors as risk factors for cryptosporidium is missing.
12. Please include how age and season were used as adjustment variables (continuous, categorical, linear).
13. LINE 147: Please provide more information about the clear structure used for the multiple comparison models. How was the structure represented in the model? Please provide references for this method. To me, this biases the model to factors that are not easily intervenable. I think it would be more clinically meaningful to focus on intervenable factors, so start with the proximal factors. If the goal is really to focus on the distal factors, I suggest framing the introduction and discussion with that goal in mind.
14. Please explain why only significant factors were left in the final model. If the goal was to understand how univariate variables change when adjusted, I suggest including all variables significant in the univariate models in the multiple variable model. If the goal is to make one final model that best predicts crypto infection, I suggest not focusing on the univariate models nor interpreting when a variable becomes not significant. If this is the aim, the final model is the focus of the interpretation.
15. Please clarify if the multiple variable model in table 3 are two final models in the methods section. (one for crypto and one fir non-crypto diarrhea)
16. Please provide more information about the imputed prevalence for the cases. A prevalence among cases after imputing for missing values?
17. Please specify how combined PAFs were combined for group level estimates in the manuscript, and not just in the supplement. PAFs cannot be added and must be summed using the following formula=(1-PAF)*(1-paf)*(1-paf). Please also add the point of how PAFs are calculated for the groups before lower level variables are added to ensure the reader know the PAF is not calculated from the final model.
18. Pleas provide more detail about the hierarchical model. Was this a 5 level model? More detail about the method used for this model is needed. Some of this is in the results section and should be moved to the method section (line 267-270). This description sounds like variables were added in stepwise without multi-level model structure. I am not familiar with this type of hierarchical modeling. Please provide a reference. Please also clarify if all variables were added, or only those significant in the univariates, or multi variable models were added. This description leads me to believe significant variables from the multivariable analyses were included, but there are some variables in the hierarchical results that are not the multivariable model. Again, see comment 15 in regards to keeping in non-significant values.
19. Please provide information about the method used for the mediation analysis in the manuscript and use a reference. I don’t think the analysis described in the supplementary materials can be considered a mediation analysis. I suggest removing this analysis.
20. I suggest stating on the outset that the population was under two and explaining why in the methods section, instead of stepwise in the results. Table 1 should be shown in the analytic sample.

Results

21. Please include reference for all variables. Reference is missing for several household environmental factors and hygiene factors in table 2.
22. Please show the counts in Table 2 and a column or in the footnotes which variables had missingness and were imputed.
23. I suggest either including p-values for all comparison in tables 2-4, or none. Currently pvalues are only included for categorical variables.
24. It would be helpful to add when you are discussing the univariate comparisons vs multiple variable model results in the text.
25. If you mention variables that were not significant, please include point estimate and 95% CIs. (line 252, 265)

Discussion

26. Include a limitations section. One large limitation is the reduce power in the crypto models. Any ‘stronger’ associations in the crypto models may be just due to power because no statistical tests can be done.
