# Supplementary material for: A comparison of risk factors for cryptosporidiosis and non-cryptosporidiosis diarrhoea: A case-case-control study in Ethiopian children
Source: PLoS Negl Trop Dis. 2022 Jun 6;16(6):e0010508. doi: 10.1371/journal.pntd.0010508 (PMC9203008; doi:10.1371/journal.pntd.0010508)
Supplement: Attachment — Submitted filename: Letter_response_to_reviewers_PLOS_NTD_REVISION.docx [file pntd.0010508.s005.docx]

**POINT-BY-POINT RESPONSE TO REVIEWERS**

**(WITH DESCRIPTION OF CHANGES MADE IN THE MANUSCRIPT AND IN SUPPLEMENTARY MATERIAL)**

**REVIEWER 1**

The author’s present a thoughtful, well written, and thorough analysis of risk factors for cryptosporidium and diarrhea in a population of children under two in Ethiopia. The manuscript applies a novel analytic strategy to covers an important and relevant public health topic. Overall, the manuscript would benefit from focus on the overall aim and additional information to the methods section.

Overall, I have never seen the case-case-control study design applied previously. I have some concerns that comparing the effect estimates from the two models are due the differences in sample size between the two models. A biostatistical should also review this paper with this specific method in mind.

**ANSWER: The issue of statistical power and precision is indeed important. A pointer to S1 Appendix (Supplementary methods - sample size considerations) has now been added to “Methods – Selection of Cases and controls” in the main manuscript. At the analysis planning stage, we consulted one of the resident biostatisticians at the University of Bergen. Furthermore, as mentioned in the revised paper, the available sample was governed by the parent study. Given that limitation, we discussed the feasibility of analyzing risk factors for cryptosporidiosis using our case and control groups, i.e., the case definition for cryptosporidiosis, and the use of mixed model logistic regression with random effect intercepts for seasonality and study enrollment site. In case-case-control studies, each case set is compared with the same control group; direct case-case comparisons are not done.**

**Having different number of children in each of the case sets does yield lower statistical efficiency than had they been of identical size, but a difference does not induce systematic errors, so we have no reason to believe that this feature undermines the credibility of our findings.**

**We agree that an any observed differences in ORs for similar exposures, between the cryptosporidiosis group and the non-cryptosporidiosis diarrhoea groups should be interpreted with caution. In addition to the already-included discussion point about sample size being too small to reliably rule out lack of exclusive breastfeeding as a risk factor, we have added an additional paragraph to the Discussion section warranting caution about strong inferences based on the observed differences in OR between Cr vs NCrD for some putative risk factors. The overall epidemiological choices (the application of an *a priori* causal framework for confounder adjustment, a hierarchical structure of this conceptual framework) involved the whole authorship group at both the planning and analysis stage of writing; several of the co-authors have experience with epidemiological methodology in general, and specific experience (including formal analysis) in causal inference applied to case-control datasets.**

More detailed suggestions are listed below:

**Introduction:**

1. The introduction could be shortened overall. Paragraph 2 does not need to include detailed conclusions about specific studies. This could be shorted to just stay the results for risk factors are inconsistent.

**ANSWER: As suggested, we have shortened and simplified the Introduction. A summary of previous risk-factor studies is now provided in the Appendix.**

2. Paragraph 3 may be better included in the methods section. A shorter description of the aims and not the rational for comparison.

**ANSWER: The justification for the use of the hierarchical conceptual framework and a case-case-control design has been moved to the Methods section, as suggested.**

3. There are a lot of comparisons in this manuscript. It is unclear if one of the main goals is to compare the epidemiologic methods here (there are 3 sets of results presented in this paper making it challenging to understand the overall conclusion). If this is the goal, I suggest limiting the comparison between crypto and non-crypto diarrhea (as mentioned above). Focusing the manuscript will help with the interpretation. If the authors want to stick with crypto vs non diarrhea comparted to non-crypto diarrhea vs non-diarrhea, I suggest removing one of the interim analyses-table 3 for example. If you believe the multilevel models are the most appropriate way to analyses these data, then why also present a mis-specified model without these levels. Choosing the primary comparison to communicate will also help focus the introduction.

**ANSWER: Thank you for highlighting to us that the reason for using a case-case-control design could have been more clearly explained. We have now emphasized this in the revised and simplified Introduction section. Furthermore, we should have clarified our use of modelling terminology, as some authors use the term “hierarchical” to refer to multilevel models (for example by the term “hierarchical modelling”), however, formal multilevel modelling is not the modelling approach used in our analysis. The word “hierarchical” instead refers to the hierarchical shape of the causal framework used to guide the modelling process, specifically the order in which adjustment variables (i.e., possible confounders) are selected and included in the multivariable models. The terminology, as we use it here, was explicitly outlined in Victora *et al*, 1997 (included in the reference list), and in later studies using a similar approach. To clarify the distinction in terminology, we removed the word “hierarchical” from the manuscript title and replaced one instance of a misleading use of the term “hierarchical models” (P 11) with the term “multivariable models”.**

**With this in mind, we would rather not remove Table 3, as rather than being interim, it is intermediate, and without the table we would need to include results (OR, CI) in the text, which would hamper the readability.**

4. Line 90: I suggest specifying the comparison in case control as you did for case to case comparison. Add the comparison “diarrhoea attributable to cryptosporidium infection compared to children with no diarrhea”

**ANSWER: Clarification added.**

**Methods:**

5. Please specify definition of diarrhea used. Any loose stools? ≥ 3 loose stools in 24 hrs?

**ANSWER: Added definition.**

6. Please specify how you defined non-diarrheal controls. No diarrhea at time of selection? No diarrhea in past 7 days? 14 days?

**ANSWER: Definition added, details of control recruitment in the appendix supplementary methods.**

7. Please specify if the non-diarrhea children had any stool testing done. Some of these may have asymptomatic infections.

**ANSWER: Stool testing of controls for asymptomatic *Cryptosporidium* infection is not part of the current analysis, as it focuses on cryptosporidiosis (i.e., symptomatic infection), but was conducted as part of the CRYPTO-POC study. Clarification added to S1 Appendix supplementary methods.**

8. Please specify how the composite was used to define crypto positive. Was a child with a positive crypto result by any method considered positive? Did it need to be 2 out of 3 tests to be crypto positive?

**ANSWER: 2 of 3, yes; details now added.**

9. Please list CT cut off values used in the methods section so the reader doesn’t have to access the reference.

**ANSWER: qIFAT and qPCR cutoffs now provided.**

10. I disagree that all variable definitions are self explanatory. Please include a brief definition of malnutrition and the cut off values in the methods section. I suggest rewording this sentence to say *other* definitions can be found in the appendix.

**ANSWER: A brief definition has now been provided in main manuscript, with additional details in S1 Appendix.**

11. Please include some information how the list of risk factors were determined in the manuscript, more detail can be kept in the supplemental materials. You could just say the list of risk factors was largely taken from key published papers. The source of perinatal factors as risk factors for cryptosporidium is missing.

**ANSWER: Perinatal risk factors are included as they are important for diarrhoea in general, and therefore considered relevant as risk factors and/or confounding factors also for cryptosporidiosis. A sentence clarifying that both cryptosporidiosis and non-cryptosporidiosis diarrhoea risk factors were investigated has been added.**

12. Please include how age and season were used as adjustment variables (continuous, categorical, linear).

**ANSWER: Specified this, with an additional pointer to S1 Appendix for details.**

13. LINE 147: Please provide more information about the clear structure used for the multiple comparison models. How was the structure represented in the model? Please provide references for this method. To me, this biases the model to factors that are not easily intervenable. I think it would be more clinically meaningful to focus on intervenable factors, so start with the proximal factors. If the goal is really to focus on the distal factors, I suggest framing the introduction and discussion with that goal in mind.

**ANSWER: The key reference (Victora *et al.* 1997) for the use of a hierarchical conceptual framework has been moved upwards in the Statistical methods paragraph. We agree with keeping a focus on factors with the potential for intervention. The hierarchical conceptual framework allows us to estimate the ORs for these proximal risk factors, while, at the same time, adjusting for risk factors that are “higher up” (i.e., more distal) in the framework. They need to be included for confounder adjustment, and their inclusion is more likely to reduce bias than to increase it. As a healthcare-centric study, it may be more likely that our study underestimates distal risk factors like poverty, SES, and maternal education, as cases and controls were recruited from the same residential areas.**

**We appreciate that we may not have made this sufficiently clear to the reader, so some clarification and justification has been added to the end of the Introduction.**

14. Please explain why only significant factors were left in the final model. If the goal was to understand how univariate variables change when adjusted, I suggest including all variables significant in the univariate models in the multiple variable model. If the goal is to make one final model that best predicts crypto infection, I suggest not focusing on the univariate models nor interpreting when a variable becomes not significant. If this is the aim, the final model is the focus of the interpretation.

**ANSWER: We see that our use of the term “final model”, and also “hierarchical multivariable analysis”, may actually confuse more than it clarifies for the reader. As you rightly point out, the main objective of this study is to obtain results as presented in Table 4, i.e., the “best estimates” for the contribution for each risk factor, while taking account both confounding and mediation from more distal levels. Our hope is to present a logical progression in the Results section, starting with univariable models, then intra-level models, and finally, a hierarchical analysis that takes all levels into account. We appreciate that we did not lay this out sufficiently clearly for the reader in the original submission and have therefore added an introductory explanation for the overall structure, just before Table 2. We have also cleaned up the terms used for this process, by clearly labelling the final analysis as a “hierarchical analysis”, as Table 4 presents results from several models, and not a single model.**

15. Please clarify if the multiple variable model in table 3 are two final models in the methods section. (one for crypto and one fir non-crypto diarrhea)

**ANSWER: As alluded to above, Table 3 (the intra-level models) represents an “intermediate step” between Table 2 and Table 4. We added an introductory explanation for this overall structure just before Table 2.**

16. Please provide more information about the imputed prevalence for the cases. A prevalence among cases after imputing for missing values?

**ANSWER: Yes, prevalence in cases and controls, respectively, after imputing missing values for exposure variables. Clarification added to footnote (details of chained random forest imputation can be found in the Methods- statistical methods sub-section).**

17. Please specify how combined PAFs were combined for group level estimates in the manuscript, and not just in the supplement. PAFs cannot be added and must be summed using the following formula=(1-PAF)*(1-paf)*(1-paf). Please also add the point of how PAFs are calculated for the groups before lower level variables are added to ensure the reader know the PAF is not calculated from the final model.

**ANSWER: We have added the point raised about the complement function and the point about distal level PAFs not being adjusted for more proximal levels to the end of the Methods section in the main manuscript.**

18. Pleas provide more detail about the hierarchical model. Was this a 5 level model? More detail about the method used for this model is needed. Some of this is in the results section and should be moved to the method section (line 267-270). This description sounds like variables were added in stepwise without multi-level model structure. I am not familiar with this type of hierarchical modeling. Please provide a reference. Please also clarify if all variables were added, or only those significant in the univariates, or multi variable models were added. This description leads me to believe significant variables from the multivariable analyses were included, but there are some variables in the hierarchical results that are not the multivariable model. Again, see comment 15 in regards to keeping in non-significant values.

**ANSWER: (Also see points above) Terminology cleaned up to avoid confusion with multilevel modelling, and reference and link for further details added to Methods. Details of how significance was used during the modelling process is provided in S1 Appendix.**

19. Please provide information about the method used for the mediation analysis in the manuscript and use a reference. I don’t think the analysis described in the supplementary materials can be considered a mediation analysis. I suggest removing this analysis.

**ANSWER: The mediation analysis used in S1 Appendix is a simple application of the traditional difference method, where regression coefficients (or, in our case, ORs) are compared between two models, one with, and another without, deliberate adjustment for mediators, taking other confounders into account. This clarification has been added to the relevant section in S1 Appendix, and a reference has been provided both in the main manuscript and in S1 Appendix. Please note that while adding these clarifications we noticed an error in S1 Appendix Table 1 (the column called “Non-mediated PAF”). We had not replaced two incorrect zeroes in one table column with their correct values; this has now been fixed. Sorry for this mistake.**

20. I suggest stating on the outset that the population was under two and explaining why in the methods section, instead of stepwise in the results. Table 1 should be shown in the analytic sample.

**ANSWER: The decision to limit the analysis to the under-2-year-olds was made a posteriori, i.e., at the stage when we had data on the prevalence of cryptosporidiosis in the whole under-5 diarrhoea case population included in the parent (CRYPTO-POC) study. We therefore feel it is more correct to include this in Results rather than Methods. However, we appreciate the point that Table 1 (the baseline data) should cover the analytic set (i.e., the under-2-year-olds), so we have modified the Table accordingly. Also, we have moved the section explaining this to the top of the Results section, to make this clearer to the reader.**

**Results**

21. Please include reference for all variables. Reference is missing for several household environmental factors and hygiene factors in table 2.

**ANSWER: The missing reference levels have been added to Table 2.**

22. Please show the counts in Table 2 and a column or in the footnotes which variables had missingness and were imputed.

**ANSWER: Table 2 footnote B now explicitly references S1 Appendix table A, which contains the breakdown of missing value numbers and proportions.**

23. I suggest either including p-values for all comparison in tables 2-4, or none. Currently pvalues are only included for categorical variables.

**ANSWER: For odds ratios we prefer 95% confidence intervals, but for tests of linear trend, where possible (e.g., for an ordered categorical variable, or a continuous variable) we use simple tests and P-levels to assess whether the dose-response relationship is statistically significant. We see that the use of a footnote at the top of the column is misleading and have specified the type of test (ordered categorical, or linear) by separate footnotes for each instance of a P-level presented in Table 2. As Table 3 and 4 contain few tests of linear trend, we have kept them as separate footnotes rather than adding a new separate column for these few values, to save space within the table.**

24. It would be helpful to add when you are discussing the univariate comparisons vs multiple variable model results in the text.

**ANSWER: Thanks for pointing this out, the first paragraph presenting univariable model results has been placed directly after the same paragraph that introduces the corresponding models, and in the subsequent paragraphs where univariable model results are followed by intra-level model results, the distinction has now been made explicit.**

25. If you mention variables that were not significant, please include point estimate and 95% CIs. (line 252, 265)

**ANSWER: The intra-level ORs with 95% CIs have been added to Results.**

**Discussion**

26. Include a limitations section. One large limitation is the reduce power in the crypto models. Any ‘stronger’ associations in the crypto models may be just due to power because no statistical tests can be done.

**ANSWER: Thank you for highlighting this. The section in Discussion where we go over caveats on interpretation of our findings and limitations is now introduced as such. We agree that the issue of power is important to address more explicitly. A pointer to S1 Appendix (Supplementary methods - sample size considerations) has now been added to “Methods – Selection of Cases and controls” in the main manuscript. In addition to the already-included discussion point about sample size being too small to reliably rule out lack of exclusive breastfeeding as a risk factor, we have added an additional paragraph has been added to Discussion warranting caution about strong inferences based on the observed differences in OR between Cr vs NCrD for some putative risk factors.**

**REVIEWER 2**

**Methods**

Reviewer #2: The objectives of study are clearly described. Study design is appropriate to address the objectives

Basic exposures and outcome variables need to define in summary and should include in the manuscript for clear understanding of the readers.

**ANSWER: A paragraph has been added to the end of the “Methods – Data collection” paragraph with a brief definition of acute malnutrition has now been provided in main manuscript, with an added pointer to details, and other variable definitions, to be found in S1 Appendix. A section called “study design”, containing some material that was previously in Results, has been moved to Methods, and a new paragraph called “Selection of cases and controls” provided.**

The samples size was not discussed. The samples size is unlikely sufficient to address all the risk factors and multivariate analysis

**ANSWER: The study size was driven by the parent study, but we appreciate that this was not stated clearly enough. A pointer to the relevant S1 Appendix (Supplementary methods - sample size considerations) has been added to “Methods – Selection of Cases and controls” in the main manuscript. We have also added an additional paragraph to Discussion warranting caution about strong inferences based on the observed differences in OR between Cr vs NCrD for some putative risk factors.**

Statistical analysis was good

The study fulfilled the criteria of ethical or regulatory requirement

**Results**

Reviewer #2: The analysis was clearly described

The results presented well

Tables and figures are sufficient quality for clarity

**Conclusions**

Reviewer #2: The conclusion is OK.

There are some limitations which need to address.

The authors addressed the data clearly.

The manuscript has public health importance. The study findings are important for researchers and policy makers.

**Editorial and Data Presentation Modifications?**

Reviewer #2: The manuscript needs minor revision.

**Summary and General Comments**

Reviewer #2: The manuscript compares the risk factors of cryptosporidiosis and non- cryptosporidiosis diarrhea cases and cryptosporidiosis vs. control without diarrhea.

The manuscript is well written and addressed case-case-control study design in children < 2 years who suffers most with cryptosporidium diarrhea and infection. This has public health importance to understand risk factors of cryptosporidiosis and to take preventive actions.

However, basic exposures and outcome variables need to define in summary and should include in the manuscript for clear understanding of the readers.

**ANSWER: See comment above where this is addressed.**

Few following comments are recommend to include in the manuscript preferably or to address.

Define diarrhea case and cryptosporidiosis in the manuscript. Risk factors for diarrhoea cases recruited in community might differ for diarrhoea cases recruited in health care settings. Similarly, diarrhoea severity might have impact on cryptosporidiosis and risk factors. However, this has not been addressed in the manuscript

**ANSWER: Case definitions for diarrhoea have now been added in the main manuscript text, with some more details on cryptosporidiosis case ascertainment added. No diarrhoea cases were recruited in the community, only the non-diarrhoea controls. Some more details have been added to Methods, under the sub-header “Selection of cases and controls”. Diarrhoeal severity may influence caregiver decisions on seeking healthcare and can therefore not be disentangled from the outcomes of healthcare-attended cryptosporidiosis and NCrD. We have now mentioned this as a limitation in Discussion.**

Moderate acute malnutrition was important predictor for cryptosporidiosis. How SAM/MAM was defined? Diarrhea and dehydration have impact on weight and to assess malnutrition status . This might overestimate MAM/SAM. This should include in discussion.

**ANSWER: Methods – Data collection now contains info on the definitions of MAM and SAM. We have now included the issue of dehydration and MAM and SAM in the limitations section of Discussion.**

What was the quantitative cut off value for diagnostic accuracy of cryptosporidiosis? How presence of other pathogens addressed in this quantitative cut off value for diagnosis of cryptosporidiosis?

**ANSWER: Quantitative cutoff levels have now been provided. Co-infections were not part of the case ascertainment or the derivation of the quantitative cutoffs. This has been clarified in the manuscript, with a reference for the parent study publication where details, and a discussion of how this may have impacted case ascertainment, was also included. To our knowledge, no study has provided evidence that the presence of other pathogens has any substantial effect on the cut-off values.**

How sufficient power was maintained to address risk factor analysis?

**ANSWER: The sample size issue has now been explicitly included in the main manuscript, with pointer to the relevant section in S1 Appendix, and has also been included as a possible limitation in the Discussion.**

**Minor comments:**

Page 2, L-50: The authors recommend following up children with previous illness. The sentence should be more conservative. This might not be feasible in low resources settings. In multivariate analysis, it is indicated as health centre visit due to illness since birth. The diarrhoea was not shown as risk factor in this analysis.

**ANSWER: Thank you for pointing out that we need to clarify this. “Previous illness” in that context encompasses all prior healthcare visits for illness, so the key point we were trying to make is that this may represent an opportunity for interventions to prevent future healthcare presentation with cryptosporidiosis. The sentence in the abstract has been rephrased accordingly. We have also rephrased the term “previous illness” as previous healthcare attendance (i.e., a proxy for previous illness), in the abstract and author summary.**

P-22, l-293: The previous illness as mentioned above.

**ANSWER: Clarified the sentence by rephrasing previous illness as previous healthcare attendance for illness.**

Page 23, L 316-317. Specify age and HIV status to indicate modest effect.

**ANSWER: Clarification has been added that the evidence for moderate effect applies to HIV-negative children aged 1-3 years.**
