# Supplementary material for: A comparison of risk factors for cryptosporidiosis and non-cryptosporidiosis diarrhoea: A case-case-control study in Ethiopian children
Source: PLoS Negl Trop Dis. 2022 Jun 6;16(6):e0010508. doi: 10.1371/journal.pntd.0010508 (PMC9203008; doi:10.1371/journal.pntd.0010508)
Supplement: Attachment — Submitted filename: Letter_response_to_reviewers_PLOS_NTD_2nd_REVISION.docx [file pntd.0010508.s006.docx]

**POINT-BY-POINT RESPONSE TO REVIEWERS**

**(WITH DESCRIPTION OF CHANGES MADE IN THE MANUSCRIPT AND IN SUPPLEMENTARY MATERIAL)**

**METHODS**

Reviewer #1: The methods well written and appropriate. A few suggestions.

Methods state that you applied quantitative cut offs to distinguish asymptomatic and symptomatic crytpo. It’s my understanding that you used quantitative results along with other test results to diagnose crypto infection. Those above the cutoff threshold still had diarrhea (symptoms), just of another cause. These are not asymptomatic crypto infections. That would be the detection of crypto in the controls.

ANSWER: As all cases had diarrhoea, we agree with the reviewer that the term “asymptomatic” should not be used here. We debated various terms to describe the group of children with diarrhoea and *Cryptosporidium* infection below the threshold deemed necessary for clinical importance (subclinical, carriage, incidental, auxiliary, background) and chose the word "incidental" (Methods: study design).

Please also clarify in the main text that no stool testing was done on controls.

ANSWER: We moved the sentence clarifying this from the Appendix to the main text (without alteration).

How did you handle age for exclusively breastfed for shorter than 6 months. This should be limited to only children who are at least 6 months of age. Based on this discussion it seems those under 6 months were grouped into the not breastfed for 6 months group.

ANSWER: We see that the wording used in the tables is misleading and have now corrected this so as to correspond to the wording used in the text (“early cessation of exclusive breastfeeding”). The definition of “early cessation of breastfeeding” has been added to the “Variables” section in the Appendix, i.e., any cessation of exclusive breastfeeding earlier than the age of 6 months (the WHO minimum recommended age for weaning or for introducing complementary foods).

We also included an explanatory bracket about this at the first mention of the “early cessation of breastfeeding” variable in the Results text section.

I suggest adding a bit more detail about the PAF mediation analysis completed in the main paper so the reader does not have to see the appendix for this method.

ANSWER: A brief summary of the method is now included at the end of the “Methods: Statistical methods” paragraph.

A note on PAFs: While going through the manuscript again, we realize that our use of the term “Total PAF” (used to represent the summary PAF that includes all risk factor levels) might be more confusing to the reader than the term ”Summary PAF”, as “Summary PAF” is this is the term that refers to the aggregate PAF of risk factors for each risk factor level. We worried that the word “total” could mislead the reader into thinking that we added up individual (or level-summary) PAFs (the sum of which can exceed 100%), when we in fact used the complement method, whereby the “summary PAF” cannot exceed 100%. We therefore replaced the term “Total PAF” with “Summary PAF for all levels” in the text (Methods L 198, Discussion L436, Table 4) and in the Appendix ("Modelling strategy and statistical methods", last paragraph).

Reviewer #2: (No Response)


**RESULTS**

-Does the analysis presented match the analysis plan?

-Are the results clearly and completely presented?

-Are the figures (Tables, Images) of sufficient quality for clarity?


Reviewer #1: Results are presented well and presented clearly.

Reviewer #2: (No Response)


**CONCLUSIONS**

-Are the conclusions supported by the data presented?

-Are the limitations of analysis clearly described?

-Do the authors discuss how these data can be helpful to advance our understanding of the topic under study?

-Is public health relevance addressed?

Reviewer #1: I think the authors overstate the strong association between crypto diarrhea and malnutrition. To some extent, this association represents the association between any diarrhea and care-seeking and malnutrition (as demonstrated by the NCrD cases). It is established that improving nutrition reduces infectious disease morbidity and the severity of that morbidity. I don’t think you can claim malnutrition and unsafe water as unique characteristics of crypto infection based on this analysis.

ANSWER: We started the Discussion by pointing out the strong association between acute malnutrition and cryptosporidiosis but now see that our phrasing runs the risk of obscuring for the reader the strong association that was also found between acute malnutrition and non-cryptosporidiosis diarrhoea. As the risk factor associations trend in the same direction, for both cryptosporidiosis and non-cryptosporidiosis diarrhoea, we agree that we should be more cautious in our wording of the observed differences. We therefore added a clarification to the introductory sentences in Discussion, and have used less assertive language about these findings in the Author summary and Abstract:Methodology/Principal findings. To give a more balanced view of both the cryptosporidiosis and non-cryptosporidiosis risk factor findings we have also included some more results (for both case groups) in Abstract:Methodology/Principal findings.

The reviewer makes a valid and important point about care-seeking. Although healthcare-presentation was mentioned in one of the paragraphs outlining limitations within the Discussion, we have now turned this into a separate and expanded paragraph to make explicit the possible influence healthcare seeking may have with the observed association between acute malnutrition and cryptosporidiosis.

Lastly, we see that our use of the word “unique” (Discussion, L 456), pertaining to the unique features of the microbe that may be related to the observed risk factor associations, is vague, and have therefore omitted this word, and also replaced the vague expression “unsafe water” with “piped water from public rather than private taps”.

Reviewer #2: (No Response)


**EDITORIAL AND DATA PRESENTATION MODIFICATIONS?**

Use this section for editorial suggestions as well as relatively minor modifications of existing data that would enhance clarity. If the only modifications needed are minor and/or editorial, you may wish to recommend “Minor Revision” or “Accept”.

Reviewer #1: (No Response)

Reviewer #2: (No Response)

**SUMMARY AND GENERAL COMMENTS**

Use this section to provide overall comments, discuss strengths/weaknesses of the study, novelty, significance, general execution and scholarship. You may also include additional comments for the author, including concerns about dual publication, research ethics, or publication ethics. If requesting major revision, please articulate the new experiments that are needed.

Reviewer #1: The author’s present a thoughtful, well written, and thorough analysis of risk factors for cryptosporidium and diarrhea in a population of children under two in Ethiopia. The manuscript applies a novel analytic strategy to covers an important and relevant public health topic and is much improved.

Reviewer #2: The Authors have sufficiently addressed earlier comments. The manuscript could be accepted after addressing few following comments.

Abstract, Background, L-34: The authors mentioned in the background that there is a "broad overlap in risk factors between cryptosporidiosis and other diarrhoeal aetiologies". However, this is not very clear how the authors addressed this issue in the manuscript.

ANSWER: Good point. We replaced this somewhat confusing attempt to explain the value of a case-case-control design, with a more specific explanation of the need for non-diarrhoea controls and leaving out of the Abstract the more detailed exposition of inconsistencies and choice of methodology. These issues were already addressed in more detail in Introduction, to which we have now also moved the sub-sentence about confounding and the use of the conceptual causal framework.

Abstract, Methodology/Principal findings: Background, L-40: The authors mentioned that "We applied quantitative cut-offs to distinguish between asymptomatic and symptomatic Cryptosporidium infection". Many of controls were positive to Cryptosporidium infection. However, I don’t see to address this in laboratory analysis section. This sentence is not consistent with the contents of manuscript in method section.

ANSWER: As all cases had diarrhoea, the use of the term “asymptomatic” is inappropriate as also pointed out by Reviewer 1. To describe the group of children with diarrhoea and *Cryptosporidium* infection below the threshold deemed necessary for clinical importance we now instead use the word "incidental" infection (in Methods:Study design). Stool testing of controls for asymptomatic *Cryptosporidium* infection is not part of the current analysis, but was conducted as part of the CRYPTO-POC study, and the sentence clarifying this has now been moved from the Appendix to the main text.

Abstract, Methodology/Principal findings: Background, L: 43-45: The statement “Side-by-side comparisons indicate that socioeconomic factors and public tap water use were more strongly associated with cryptosporidiosis than with non-cryptosporidiosis diarrhoea” is likely not consistent as per table 3 and table 4 especially for socioeconomic factors.

ANSWER: This statement refers to the PAF estimates in Table 4, and the “socioeconomic factors” refers to low maternal education specifically. We have modified the last sentence in Abstract:Methodology/Principal findings to make this clearer.

Selection of cases and controls, page 5, L: 122-123: The definition of diarrhea or dysentery needs to be specific. Was the diarrhoea acute or chronic? If diarrhoea is acute, how was it defined to differentiate from chronic diarrhoea?

ANSWER: The diarrhoea and dysentery definitions have been added, as well as a clarification that both acute, prolonged, and persistent (which also encompasses chronic) diarrhoea cases were included.

Fig 1, Level 5: Minor editions: “Previous illness” is termed as “previous healthcare attendance” in revised manuscript. It is recommended to make it consistent throughout the manuscript

ANSWER: Good point. We used the catch-all term “previous illness history” in the tables, of which previous healthcare attendance is one of several variables. In the conceptual causal framework figure, we have now changed the labelling from “Level 5: Breastfeeding, anthropometric status, and previous illness” to “Level 5: Breastfeeding, anthropometric status, previous illness history” to make the terminology more consistent across tables, figures, and text.

Table 1, page 11: The percentages have not corrected/updated for age in months in controls, Cryptosporidiosis and non-cryptosporidiosis.

ANSWER: Thank you very much for noticing this error after updating the manuscript table, we have now corrected the Table 1 percentage figures (in brackets).

Table A in S1 Appendix: The number of animal owned (n=1572) in Tab A in S1 Appendix is confusing. The number is more than the denominator (n=725). Are the authors indicating households owned animals or the total number of animals all the household of study participants?

ANSWER: We see that the row header “Animals owned by the household” is imprecise and can lead to confusion. For each row of animals (e.g., “cattle”) or animal groupings (e.g., “any even-toed ungulate”) there is a binary yes/no response category, i.e., ownership of any number of animals of the given type qualifies as “exposed”. We have changed the row header to “Animals owned by the household (household ownership of ≥1 animal of the stated type)” to make this clear.

**MINOR EDITIONS:**
Introduction, page 4, Line 78: Add “.” at the end of the sentence.
Introduction, page 4, L-96: Delete “-“

ANSWER: Great, thank you for noticing those errors - corrections made accordingly.

General: Thank you again to both reviewers for the their valuable questions and helpful comments to our manuscript.
